# *Bifidobacterium infantis* Promotes Foxp3 Expression in Colon Cells *via* PD-L1-Mediated Inhibition of the PI3K-Akt-mTOR Signaling Pathway

**DOI:** 10.3389/fimmu.2022.871705

**Published:** 2022-07-04

**Authors:** Linyan Zhou, Ying Xie, Yan Li

**Affiliations:** Gastroenterology department, ShengJing Hospital of China Medical University, Shenyang, China

**Keywords:** IBD, Bifidobacterium Infantis, colon cell, signaling pathway, Foxp3

## Abstract

**Aim:**

Our objective was to investigate whether Bifidobacterium infantis inhibits PI3K-Akt-mTOR signaling and upregulates Foxp3 expression through PD-L1 and to explore the possible mechanism of action of B. infantis in cellular immunosuppression.

**Method:**

The effects of B. infantis supernatant on PD-L1, PD-1, Foxp3, and the PI3K-Akt-mTOR signaling pathway were observed by culturing HCT-116 cells. Simultaneously, the effects of blocking PD-L1 on PD-1, on Foxp3 protein and mRNA, and on the PI3K-Akt-mTOR signaling pathway protein were observed.

**Results:**

B. infantis supernatant was able to upregulate the protein and mRNA expression of PD-L1 and Foxp3 and downregulate the phosphorylated protein expression of PI3K, Akt, and mTOR (P < 0.05); however, for PI3K, Akt, and mTOR, there was no change in the total protein expression. After the blocking of PD-L1, the stimulatory effect of B. infantis supernatant on Foxp3 and the inhibitory effect on the phosphorylated protein expression of PI3K, Akt, and mTOR were weakened.

**Conclusion:**

B. infantis may inhibit the PI3K-Akt-mTOR signaling pathway and promote the expression of Foxp3 through PD-L1, which may be a target *via* which B. infantis exerts its immunosuppressive effect.

## Introduction

Inflammatory bowel disease (IBD) is a disease of the digestive system that seriously endangers the health of the sufferer (1). IBD often damages organs outside the digestive tract, including the eyes, skin, mucosa, joints, liver, and pancreas. Patients suffering from IBD usually exhibit gastrointestinal bleeding, fistula, perforation, cancer, and other complications, which can affect their quality of life and ability to work (2). These conditions endanger the health of the patients and impose an economic burden on their families (3, 4). It is therefore important to find an effective treatment for this condition.

Forkhead box protein 3 (Foxp3) is a regulatory T cell (Treg) marker that exerts an immunosuppressive effect (5). Foxp3^+^Tregs, which stably express Foxp3, are primarily produced in the thymus and move to the periphery where they exert an immunosuppressive effect, while Foxp3^−^Tregs have no such function (6). Therefore, Foxp3 plays a key role in immunosuppression and cell stability. Our research group previously found that *Bifidobacterium infantis* (*B. infantis*) can promote the expression of Foxp3 in Tregs by upregulating PD-L1, thereby promoting cell proliferation and improving the anti-inflammatory function of these cells, so as to inhibit the intestinal immune response, and this provided new clues for the immunosuppressive treatment of IBD (7). However, the mechanism by which *B. infantis* promotes Foxp3 expression remains to be evaluated.

The PI3K-Akt-mTOR signaling pathway is involved in cell growth, differentiation, and apoptosis and shows crosstalk with other important signal transduction pathways (8). The differentiation and proliferation of initial T lymphocytes depends on the activation of the Akt-mTOR signaling pathway (9). Activation of this signaling pathway can inhibit the differentiation of Tregs (10), while its inhibition promotes an increase in Foxp3 expression and affects the Treg counts in animals. Francisco et al. (11) found that PD-L1-negative antigen-presenting cells induce the transformation of CD4^+^T cells into Foxp3^+^Tregs to a minimal extent, suggesting the importance of PD-L1 in Treg differentiation. Simultaneously, they found that PD-L1 can antagonize the Akt-mTOR signaling pathway, which provided a new idea for studies on the mechanism by which PD-L1 regulates Tregs. However, there are few studies on the identity of the signaling pathway modulated by *B. infantis*, which triggers the immune response.

Based on the above theory, we speculated that *B. infantis* could increase the expression of Foxp3 by upregulating PD-L1 and inhibiting PI3K-Akt-mTOR signaling. Several studies have shown that Foxp3, PD-L1, and PD-1 are expressed in HCT-116 cells (12–14). Therefore, we chose this cell line as a model to study whether *B. infantis could* inhibit PI3K-Akt-mTOR signaling and upregulate Foxp3 expression through PD-L1, so as to further explore the possible mechanism by which *B. infantis* exerts an immunosuppressive effect.

## Methods

### Materials

The HCT-116 cell line was purchased from ATCC, USA. *B. infantis* CGMCC No. 0313-2 (Batch No. 2017012) was provided Kexing Biological Products Co., Ltd, Shandong, China. Anti-PD-L1 was purchased from BIOX cell, New Hampshire, USA. Protein marker (m000624) was purchased from Kingsley Biotechnology Co., Ltd, Nanjing, Jiangsu, China. Polyvinylidene fluoride (PVDF) membranes were purchased from the GE company, Fairfield, Connecticut, USA. GAPDH antibody, phospho-PI3K antibody, and phospho-mTOR antibody were purchased from Abcam, USA. PD-L1, PD-1, Foxp3, AKT1, and mTOR antibodies were purchased from Proteintech, USA. PI3K, phospho-Akt, PTEN, and phospho-PTEN antibodies were purchased from CST, USA. Sensitive chemiluminescence solution was sourced from Millipore, USA. PrimeScript RT reagent kit with gDNA Eraser was purchased from TaKaRa Bio, Japan. The qRT-PCR kit SYBR Premix Ex Taq II (TLI RNaseH Plus) was from TaKaRa. TPY agar medium and TPY broth were purchased from Qingdao Haibo Biotechnology Co., Ltd, China. All primers were purchased from China Bio Engineering Co., Ltd. The turbidimetric tube was purchased from China Wenzhou Kangtai Biotechnology Co., Ltd.

### Cell Culture

HCT-116 cells were cultured in McCoy’s 5A medium containing 10% fetal bovine serum, 1% penicillin-streptomycin, and double antibody at 37°C and 5% carbon dioxide. The culture medium was changed every 24–48 hours. When the confluency reached ~80%, the cell line was passaged.

### Obtaining the Supernatant of *B. infantis*

*B. infantis* lyophilized powder was diluted in a sterile solution. A loopful of liquid bacterial culture was inoculated with the bacterial liquid and then inoculated on the TPY plate medium according to the three-zone marking method under a flame. The culture dish was placed in an inverted position in a round-bottom three-dimensional anaerobic culture bag and incubated at 37°C for 48 h.

### Identification of *B. infantis*

Colony morphology was observed on the culture medium and attention was paid to no presence of any miscellaneous bacteria. The anaerobic indicator color change was checked to ensure the anaerobic state. A single colony was selected, coated on the slide, dried, and fixed. After staining and decolorization, the morphology of the bacteria was observed under an oil immersion objective and photographed.

### Preparation of *B. infantis* Supernatant

*B. infantis* colonies were collected and inoculated in sterile normal saline. The concentration was adjusted to 3 × 10^9^ CFU/ml in turbidimetric tubes and centrifuged at 5000 rpm. *B. infantis* was cultured in TPY broth at 37°C for 96 h. After centrifugation, the supernatant was filtered using a 0.22-μm pore filter to confirm that the supernatant was sterile. Aseptic conditions were observed throughout the procedure.

### Cell Modeling and Grouping

Cells in the logarithmic growth phase were seeded in 6-well plates and cultured for 12 h. After confirming adherence, the cells were cultured for 24 h with the medium. In total, we tested five groups: control group, TPY group, TPY+*B. infantis* group, TPY+*B. infantis *+PD-L1 Blockade group, and TPY+PD-L1 Blockade group.

### Immunohistochemistry

After immobilization with 4% paraformaldehyde, samples were blocked using endogenous peroxidase and nonspecific antigens. The primary antibodies were used to probe the samples at the following dilutions: PD-1, 1:200 (Proteintech, 18106-1-AP); PD-L1, 1:50 (Proteintech, 17952-1-AP); and Foxp3, 1:300 (Proteintech, 22228-1-AP). After incubation with the secondary antibody, horseradish labeling, DAB color development, restaining, and dehydration, three low-power microscope fields were randomly selected to investigate protein localization and detection.

### Western Blotting

The total cell proteins were extracted, the protein concentration was determined, and protein samples of 40 μg were prepared. The proteins were separated *via* 60-V constant pressure electrophoresis, membrane transferred at 100 V, and blocked using 2.5% BSA. The PVDF membrane was developed in a dark room to detect the protein bands. Gelpro software was used to analyze gel image results and perform protein quantitative analysis. The specific antibody dilutions were as follows:

**Table:** 

Antibody	Dilution concentration	Company	Catalog Number
PD-L1	1:750	Proteintech	17952-1-AP
PD-1	1:500	Proteintech	18106-1-AP
Foxp3	1:1000	Abcam	ab54501
PI3K	1:1000	CST	4257
phospho-PI3K	1:1000	Abcam	ab182651
AKT1	1:1000	Proteintech	10176-2-AP
phospho-Akt	1:2000	CST	4060
PTEN,	1:1000	CST	51-2400
phospho-PTEN	1:1000	CST	9551
mTOR	1:500	Proteintech	20657-1-AP
phospho-mTOR	1:10000	Abcam	ab109268
GAPDH	1:10000	Abcam	ab181602

### Quantitative Real-Time Fluorescent Reverse Transcription Polymerase Chain Reaction (qRT-PCR)

qRT-PCR was carried out after RNA extraction and purity evaluation, reverse transcription, and cDNA synthesis and purity evaluation. The primer sequences are shown in **Table 1**.

**Table 1:** Primer sequence.

Primer	Sequence
PDL1f	GCTGCACTAATTGTCTATTGGG
PDL1r	CACAGTAATTCGCTTGTAGTCG
PD-1f	GACTGCCGCTTCCGTGTCAC
PD-1r	GAGGTAGGTGCCGCTGTCATTG
FOXP3f	CTCTTCTTCCTTGAACCCCAT
FOXP3r	CTGGAGGAGTGCCTGTAAG
GAPDHf	CAGGAGGCATTGCTGATGAT
GAPDHr	GAAGGCTGGGGCTCATTT

### Statistical Analysis

SPSS 23.0 and GraphPad 7.0 statistical software were used for analysis. The measurement data are expressed as mean ± standard deviation. Analysis of variance was used for comparison between groups, with P < 0.05 regarded as statistically significant.

## Results

### Localization and Expression of PD-L1, PD-1, and Foxp3 in Colon HCT-116 Cells

**Figure 1:**
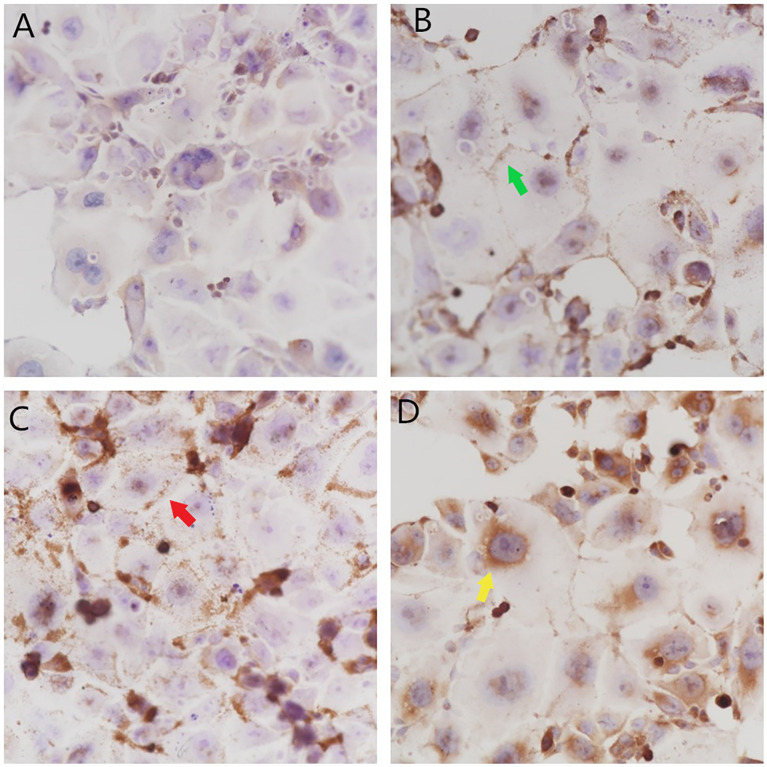
Localization and expression of PD-L1, PD-1, and Foxp3 in colon HCT-116 cells. **(A)** The negative control group (× 400) showed no obvious brown granule deposition. **(B)** The PD-L1 group (× 400) showed brown granule deposition on the cell membrane 
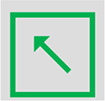
 and a small amount of brown granule deposition in the cytoplasm. **(C)** The PD-1 group (× 400) showed a large amount of brown granule deposition on the cell membrane 
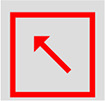
 and a small amount of brown granule deposition in the cytoplasm. **(D)** The Foxp3 group (× 400) showed a large amount of brown granule deposition in the nucleolus and on the nuclear membrane
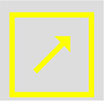
.

In the negative control group, the nuclei showed dark blue staining, cell membranes showed no obvious staining, and the cytoplasm showed light blue staining, with a small amount of uneven light brown particles, as shown in **Figure 1A**. After PD-L1 positioning, the nucleus was stained dark blue, a few brown granules were found in several nuclei, the cell membrane was stained with dark brown granules, the cytoplasm was uniformly stained light blue, and a few yellow granules were scattered, as shown in **Figure 1B**. After PD-1 positioning, the nucleus was stained dark blue, brown granules were deposited in individual nuclei, a large number of dark brown granules were observed in the cell membrane, the cytoplasm was uniformly stained light blue, and yellow granules were observed, as shown in **Figure 1C**. After Foxp3 positioning, the nucleus was stained dark blue, a large number of brown granules were found in the nucleolus and nuclear membrane, almost no dark brown granules were found in the cell membrane, the cytoplasm was uniformly stained light blue in the middle, and yellow granules were found in the cytoplasm of the individual cells, as shown in **Figure 1D**.

### Effect of *B. infantis* Supernatant on PD-L1 Expression

**Figure 2:**
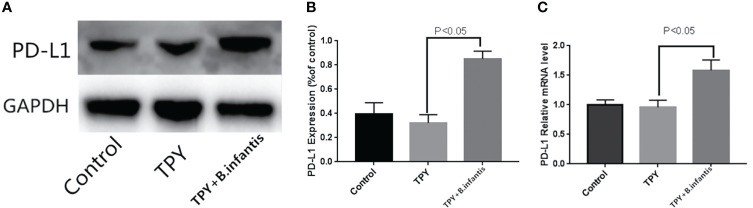
Effect of *Bifidobacterium infantis* supernatant on PD-L1 expression. **(A)** PD-L1 protein expression band. **(B)** Differences in PD-L1 protein expression. **(C)** Differences in PD-L1 mRNA expression. The data in the figure are expressed as mean ± standard deviation, and the sample comparison among the groups was conducted using one-way ANOVA. P < 0.05 was considered statistically significant.

*B.infantis* impoved the expression of PD-L1.There was no significant difference in PD-L1 mRNA or protein expression between the TPY and control groups. Compared with that in the TPY group, the expression of the PD-L1 protein and mRNA in the TPY+*B. infantis* group was significantly higher (P < 0.05), as shown in **Figure 2**.

### Effect of *B. infantis* Supernatant on PD-1 Expression After Blocking PD-L1

**Figure 3:**
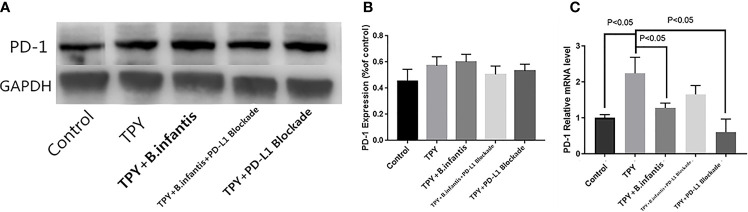
Effect of *Bifidobacterium infantis* supernatant on PD-1 expression. **(A)** PD-1 protein expression band. **(B)** Differences in PD-1 protein expression. **(C)** Differences in PD-1 mRNA expression. The data in the figure are expressed as mean ± standard deviation, and the sample comparison among the groups was conducted using one-way ANOVA. P < 0.05 was considered statistically significant.

*B.infantis* didn’t promote or lead to the protein expression of PD-1, but inhibited the RNA expression of PD-1 interestingly. TPY broth increased the RNA expression of PD1, and this promotion was weakened after blocking PD-L1. There was no significant difference in the expression of PD-1 protein between the TPY group and control group (P = 0.11). Similarly, there was no change in PD-1 protein expression between the TPY and TPY+*B. infantis* groups (P = 0.90). There was no significant difference in PD-1 protein expression between the TPY+*B. infantis* and TPY+*B. infantis*+PD-L1 Blockade groups (P = 0.13). Compared with that in the TPY group, there was no significant difference in the expression of the PD-1 protein in the TPY+PD-L1 Blockade group (P = 0.14), as shown in **Figure 3**.

The expression of PD-1 mRNA differed from the protein expression. Compared with that in the control group, the expression of PD-1 mRNA in the TPY group was significantly higher (P < 0.05), while compared with that in the TPY group, the expression of PD-1 mRNA in the TPY+*B. infantis* group was significantly lower (P < 0.05). After adding the PD-L1 blocker, the expression of PD-1 mRNA in the TPY group also decreased significantly (P < 0.05), as shown in **Figure 3**.

### Effect of *B. infantis* Supernatant on Foxp3 Expression After the Blocking of PD-L1

**Figure 4:**
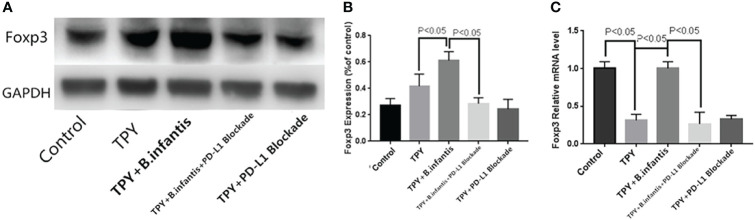
Effect of *Bifidobacterium infantis* supernatant on Foxp3 expression. **(A)** Foxp3 protein expression band. **(B)** Differences in Foxp3 protein expression. **(C)** Differences in Foxp3 mRNA expression. The data in the figure are expressed as mean ± standard deviation, and the sample comparison among the groups was conducted using one-way ANOVA. P < 0.05 was considered statistically significant.

TPY broth dramatically decreased Foxp3 RNA, but had no significant effect on its protein. The addition of *B.infantis* significantly increased the expression of Foxp3. However, after blocking PD-L1, the promotion effect was also neutralized. Compared with that in the control group, Foxp3 protein expression in the TPY group showed no significant change (P = 0.13). Compared with that in the TPY group, Foxp3 protein expression in the TPY+*B. infantis* group was significantly higher (P < 0.05). Compared with that in the TPY+*B. infantis*+PD-L1 Blockade group, Foxp3 protein expression in the TPY+*B. infantis* group was significantly lower (P < 0.05). There was no significant difference in Foxp3 protein expression between the TPY group and the TPY+PD-L1 Blockade group (P = 0.14), as shown in **Figure 4**.

Compared with that in the control group, Foxp3 mRNA expression in the TPY group was significantly higher (P < 0.05). Compared with that in the TPY group, the expression level of Foxp3 mRNA in the TPY+*B. infantis* group was significantly higher (P < 0.05). After adding the PD-L1 blocker, the expression of Foxp3 mRNA in the TPY+*B. infantis* group decreased significantly (P < 0.05). There was no significant difference in Foxp3 mRNA expression between the TPY group and TPY+PD-L1 Blockade group (P = 0.97), as shown in **Figure 4**.

### Effect of *B. infantis* Supernatant on the PI3K-Akt-mTOR Pathway After the Blocking of PD-L1

**Figure 5:**
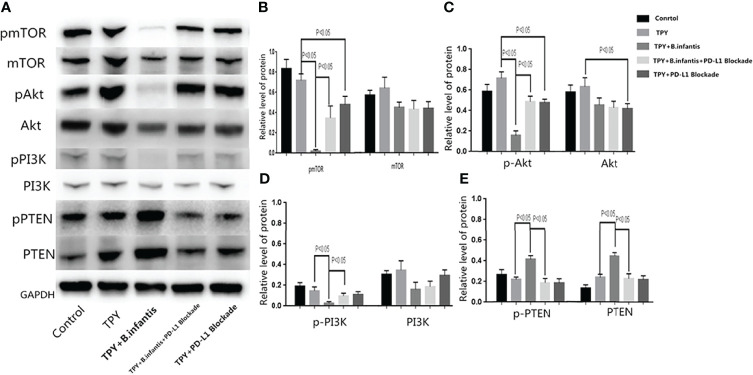
Effect of ***B.*** *infantis* supernatant on the PI3K-Akt-mTOR pathway after the blocking of PD-L1. **(A)** mTOR, Akt, PI3K, PTEN phosphorylated protein and total protein expression. **(B)** A statistical chart showing the p-mTOR and mTOR protein expression difference. **(C)** A statistical chart showing the p-Akt and Akt protein expression difference. **(D)** A statistical chart showing the p-PI3K and PI3K protein expression difference. **(E)** A statistical chart showing the p-PTEN and PTEN protein expression difference. The data in the figure are expressed as mean ± standard deviation, and the sample comparison among the groups was conducted using one-way ANOVA. P < 0.05 was considered statistically significant.

*B.infantis* had inhibitory effects on p-MTOR, p-Akt and p-PI3K, and this inhibitory effect disappeared after blocking PD-L1. The addition of *B.infantis* did not change mTOR, Akt and PI3K. The effect of *B.infantis* on p-PTEN and PTEN was opposite to that of PI3K-Akt-mTOR pathway. Compared with that in the TPY group, the whole phosphorylated protein expression of PI3K, Akt, and mTOR in the TPY+*B. infantis* group was significantly lower (P < 0.05), while the total protein content of PI3K, Akt, and mTOR showed no significant changes (P values of 0.186, 0.178, and 0.19, respectively). Compared with that in the TPY group, The phosphorylated protein and total protein PTEN contents were significantly higher (P < 0.05). The phosphorylated protein expression of mTOR, Akt, and PI3K in the TPY+*B. infantis* group was significantly higher than that in the TPY+*B. infantis*+PD-L1 Blockade group. After adding the PD-L1 blocker, the phosphorylated protein expression of mTOR and Akt and the total protein expression of Akt in the TPY group decreased significantly (P < 0.05), while the other proteins showed no significant change (**Figure 5** **)**.

## Discussion

Probiotics are defined as living microorganisms that when given in sufficient quantities, can provide health benefits to the host (15). At present, the use of probiotics in the treatment of digestive system diseases is a promising alternative and adjuvant therapy (16) (17). Previous studies have shown that probiotics can play a therapeutic role in the regulation of immunity, gut flora composition, and protein and lipid metabolism (18, 19). We found that *B. infantis* mediated Foxp3 expression through PD-1/PD-L1 pathway, which promoted Tregs differentiation and improved IL-10 and TGF- β 1 expression, so as to reduce the immune and inflammatory response of mice with inflammatory bowel disease. In order to study how *B. infantis* transmits the signal to Foxp3, we designed the experiment *in vitro*. Recent studies have shown that supernatants of cultured probiotics, such as the supernatant of *B. infantis*, can exert the same beneficial effects as live bacteria, inhibiting the growth of pathogenic bacteria and improving epithelial barrier function and immune regulation (20, 21). However, the exact mechanism remains to be clarified.

The PI3K-Akt-mTOR signaling pathway is involved in cell proliferation, differentiation, apoptosis, and other types of cell regulation (22). When the ligand binds to the cell membrane receptor, the receptor activates phosphatidylinositol 3-kinase (PI3K), promoting the activation of a second messenger, further activating protein kinase B (Akt) and regulating cell proliferation and survival by phosphorylating mammalian target of rapamycin (mTOR) (23). *PTEN* is a tumor suppressor gene. As a key phosphatase, it can inhibit the formation of PI3K and reduce Akt activation and its downstream signal transduction (24). Our research showed that TPY exerted no statistically significant change in the PI3K-Akt-mTOR pathway proteins. We also found that the supernatant of *B. infantis* could reduce the protein expression of p-mTOR, p-Akt, and p-PI3K; however, it exerted no significant effect on the total protein content of mTOR, Akt, and PI3K, which may be due to the inhibition of PI3K-Akt-mTOR signaling/expression by fermentation products of *B. infantis*. *B. infantis* supernatant could significantly upregulate PD-L1 expression. Further, it was found that TPY broth could increase PD-1 mRNA expression but had no significant effect on the PD-1 protein. *B. infantis* supernatant could reduce PD-1 mRNA expression but had no effect on the PD-1 protein. We speculated that some components of the TPY broth could promote the gene transcription of PD-1, while *B. infantis* could counteract this effect. However, the specific mechanism remains to be studied further.

In recent years, studies have found that PD-L1 can inhibit PI3K-Akt-mTOR signaling (25, 26). PD-L1 promotes the high expression of PTEN, which can antagonize the PI3K-mediated signal, which promotes cell metabolism, growth, proliferation, and survival (27). The PI3K-Akt-mTOR signaling pathway is closely related to IBD (28). Studies have found that activation of this signal transduction pathway can activate NF-κB, which, in turn, activates TNF-α, IL-1, IL-6, and other inflammatory factors and mediators, thus amplifying and sustaining the inflammatory response. *Lactobacillus* can reduce the phosphorylated Akt levels in rat gastric sphincter and may further inhibit the pro-inflammatory factor IL-6, thus promoting TGF-β synthesis, so as to regulate the immune response (29). Studies have shown that *B. infantis* can inhibit the inflammatory response through the PI3K-Akt signaling pathway. *B. infantis* supernatant can regulate dendritic cell function by regulating mitogen activated protein kinase (MAPK), glycogen synthase kinase-3 (GSK3), and PI3K to different degrees through this cell pathway (30).

To confirm whether the supernatant of *B. infantis* affects PD-1 expression, the PI3K-Akt-mTOR signaling pathway, and Foxp3 by increasing PD-L1, we added the PD-L1 blocker to colon cells *in vitro*. The results showed that when PD-L1 was blocked, the *B. infantis* supernatant had no significant effect on the protein or mRNA expression of PD-1. The relationship between PD-L1 and PD-1 as ligands and receptors. We analyzed the blocking of ligands did not affect the changes of the receptors caused by *B. infantis*, while PD-L1 significantly inhibited the expression of PD-1 mRNA but did not change PD-1 protein levels. The protein expression of p-mTOR, p-Akt, and p-PI3K increased significantly in the PD-L1 antagonist group; however, the total protein expression of mTOR, Akt, and PI3K remained unchanged, while the phosphorylation and total PTEN expression decreased significantly. We believe that the action on PD-L1 is the main mechanism by which *B. infantis* exerts its immunosuppressive effect. After blocking PD-L1, the expression of PTEN decreased. Furthermore, the inhibition of PI3K-Akt-mTOR signaling was reduced. Compared with that in the TPY group, the expression of p-mTOR, p-Akt, and total Akt protein decreased after the PD-L1 blocker was added, suggesting that the PD-L1 protein itself may stimulate the activation of p-mTOR, p-Akt, and total Akt protein. We infer that *B. infantis* first promoted the massive expression of PD-L1, the combination of PD-L1 and PD-1, then inhibited the phosphorylation expression of PI3K, Akt and mTOR, finally promotes the translation of Foxp3, and then might induce Treg differentiation. However, after PI3K phosphorylation was inhibited, why did the expression of Akt and mTOR also change? We suspect that *B. infantis* might directly affect Akt and mTOR phosphorylation through PD-L1, rather than through the PI3K-Akt-mTOR signaling. However, the specific underlying mechanism requires further study.

*B. infantis* supernatant promoted the expression of Foxp3; however, the expression of Foxp3 mRNA and protein significantly decreased after blocking PD-L1, suggesting that PD-L1 is the main intermediate pathway of the *B. infantis* supernatant to promote the expression of Foxp3. TPY broth decreased the expression of Foxp3 mRNA; however, it had no significant effect on the Foxp3 protein. We speculated that some component of the TPY broth downregulated the transcription of Foxp3 but did not affect the translation process.

## Conclusion

**Figure 6:**
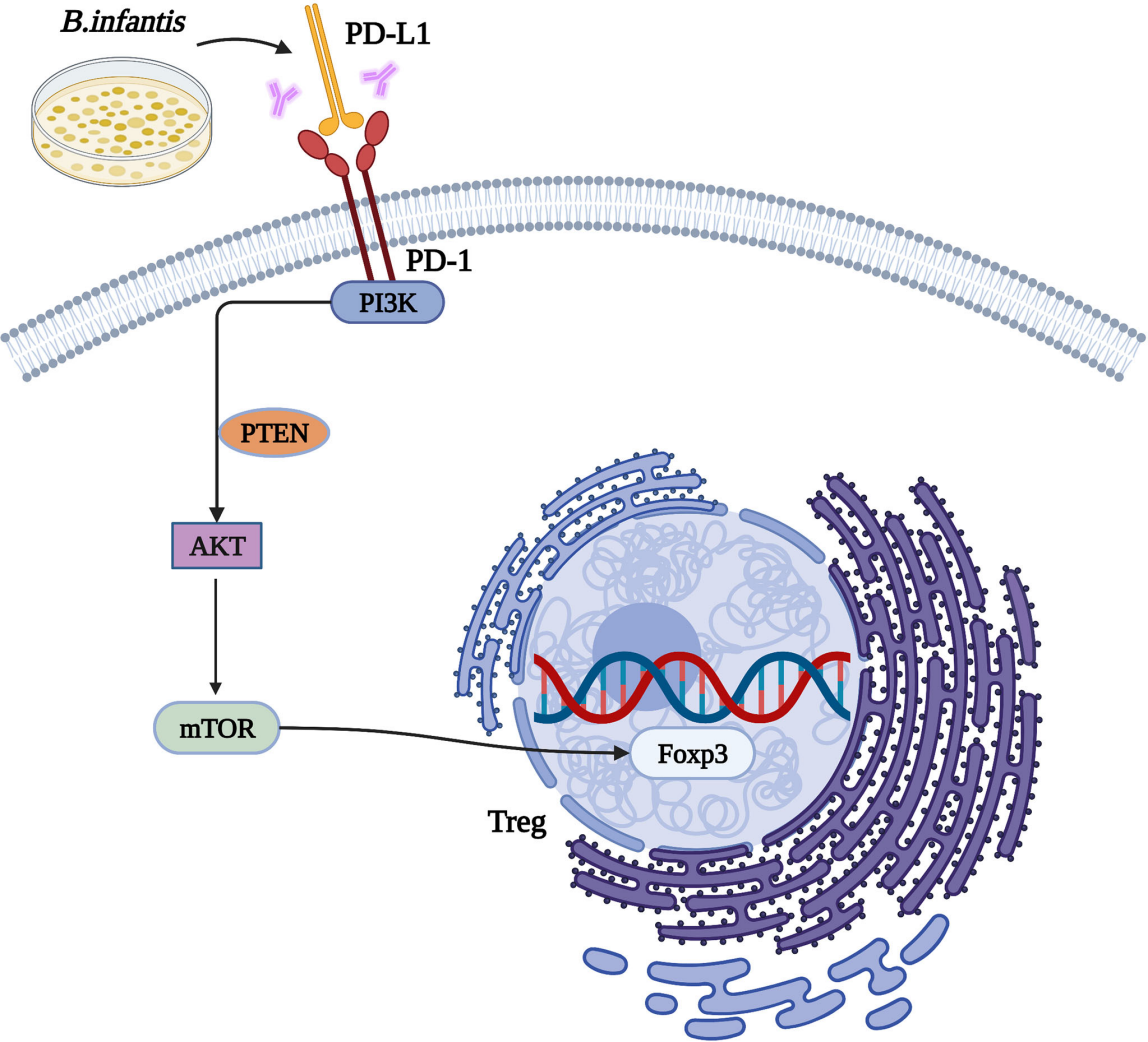
The mechanism by which *Bifidobacterium infantis* mediates the PD-1/PD-L1 pathway by inhibiting Foxp3 expression.

In conclusion, we found that *B. infantis* could inhibit PI3K-Akt-mTOR signaling and promote the expression of Foxp3 through PD-L1, so as to exert an immunosuppressive effect (see **Figure 6**). However, the specific mechanism needs to be explored further.

## Data Availability Statement

The original contributions presented in the study are included in the article/supplementary material. Further inquiries can be directed to the corresponding author.

## Author Contributions

LZ wrote manuscript, processed the samples, analyzed the raw data, obtained funding, and approved the final version of the manuscript. LZ and YX processed the samples, complete the experiment together. YL conceived the study protocol, critically revised the manuscript, and approved its final version. All authors contributed to the article and approved the submitted version. All authors contributed to the article and approved the submitted version.

## Funding

This study was supported by the Doctoral Start-up Foundation of Liaoning Province (Contract No. 2021-BS-114).

## Conflict of Interest

The authors declare that the research was conducted in the absence of any commercial or financial relationships that could be construed as a potential conflict of interest.

## Publisher’s Note

All claims expressed in this article are solely those of the authors and do not necessarily represent those of their affiliated organizations, or those of the publisher, the editors and the reviewers. Any product that may be evaluated in this article, or claim that may be made by its manufacturer, is not guaranteed or endorsed by the publisher.
